# Ensemble-Based Interpretations of NMR Structural Data to Describe Protein Internal Dynamics

**DOI:** 10.3390/molecules180910548

**Published:** 2013-08-30

**Authors:** Annamária F. Ángyán, Zoltán Gáspári

**Affiliations:** Faculty of Information Technology, Pázmány Péter Catholic University, Budapest 1083, Práter street 50/A, Hungary; E-Mail: gaspari.zoltan@itk.ppke.hu

**Keywords:** dynamic structural ensembles, protein NMR spectroscopy, protein mobility, molecular dynamics simulation, ensemble generation

## Abstract

NMR spectroscopy is the leading technique to characterize protein internal dynamics at the atomic level and on multiple time scales. However, the structural interpretation of the observables obtained by various measurements is not always straightforward and in many cases dynamics-related parameters are only used to “decorate” static structural models without offering explicit description of conformational heterogeneity. To overcome such limitations, several computational techniques have been developed to generate ensemble-based representations of protein structure and dynamics with the use of NMR-derived data. An important common aspect of the methods is that NMR observables and derived parameters are interpreted as properties of the ensemble instead of individual conformers. The resulting ensembles reflect the experimentally determined internal mobility of proteins at a given time scale and can be used to understand the role of internal motions in biological processes at atomic detail. In this review we provide an overview of the calculation methods currently available and examples of biological insights obtained by the ensemble-based models of the proteins investigated.

## 1. Introduction

Nuclear magnetic resonance spectroscopy is the method of choice for structural investigation of proteins and peptides in solution. The diversity of described pulse sequences and the availability of analysis tools and automated structure refinement protocols offer convenient solutions for the most common structure-related problems. However, the true strength of today’s biomolecular NMR is its ability to report on the internal dynamics of the molecules at the atomic level and on multiple time scales [1]. Although the dynamic nature of proteins was recognized long before the first dynamics measurements, results obtained with NMR spectroscopy led to the emergence of the current paradigm of protein action where internal dynamics is a key factor in determining and tuning molecular function [2,3].

Despite this dynamics-based view, structural representations of proteins where a single conformer or a number of highly similar ones are shown and used for biological interpretations are still prevalent. The success of solution-state structure determination of proteins is often measured with the similarity to a single X-ray structure [4]. However, today there are a number of techniques available to generate ensemble-based representations of protein structures where the diversity of the ensemble reflects the experimentally observed internal dynamics. Such ensembles can successfully be used to understand biochemical mechanisms at the atomic level [5].

Intrinsically disordered proteins (IDPs) comprise the most prevalent examples of systems where the single-conformer approach is not feasible. Their extreme flexibility compared to globular proteins pointed to the need of ensemble interpretations of experimental data from the earliest systematic structural studies of such systems [6]. However, although the highly dynamical nature of such systems gives rise to previously unknown biochemical mechanisms, such as negative coupling between distinct partner binding sites [7], their characterization at the atomic level is often a challenge.

In this review we outline these methods with emphasis on those directly reflecting the ensemble-based nature of NMR data. We argue that the resulting ensembles are better models of protein structures than “static” ones and provide examples of conclusions that could be drawn with the use of ensemble representations.

## 2. Models of Protein Structures

An important point to make right at the beginning is that all protein structures we encounter are models [8]. This must be stressed as the term “model” in structural biology is usually restricted to structures that are generated without any direct experimental information (e.g., homology models). However, in the strict sense, even structures that were obtained with the use of extensive experiment-derived parameters are models of the actual entities investigated. Thus, we have to be aware that they can have different types of errors and might be suitable for one purpose but not another. The precision of the structure comes from the experimental errors, while its accuracy reflects its correspondence to “reality” [8]. Accuracy can only be assessed by independent measurements, *i.e.*, by determining the same structure both by X-ray and NMR, but also different types of NMR observables can provide independent sources of information. Thus, an accurate model should be consistent with a high number of experimental parameters.

In the conventional way of NMR structure determination (termed SCR for single-conformer refinement below), a number of conformers are generated with the aim that each of them should correspond to as many experimental parameters as possible, ensured by the application of restraints derived from experimental measurements [9]. Thus, the best ones are selected, meaning those that exhibit the lowest number of restraint violation and are similar to each other, *i.e.*, represent the same structure. It should be stressed here that structure determination protocols use force field-type parametrization to ensure the adequate geometry of the structures generated, thus, the balance between geometric and restraint terms is a key issue in such protocols. In contrast to the approximation inherent in the SCR methodology, the NMR tube contains on the order of 10^16^–10^17^ dynamically fluctuating molecules, thus, each measured parameter represents the time and ensemble average of high number of conformers [10,11]. In practice, this means that we can not necessarily expect that there is a single conformer in the tube that simultaneously fulfills all experimental restraints. This notion was formulated for H-D exchange protection factors [12] but is of more general significance.

A possible solution of this apparent contradiction is to use ensemble-based representations, where we only require that the averaged NMR parameters are consistent with the experimental data. These ensemble-based representations, termed dynamic structural ensembles below, comprise a novel kind of models of protein structures and pose some novel challenges in their validation and interpretation, which will be discussed further below.

**Figure 1:**
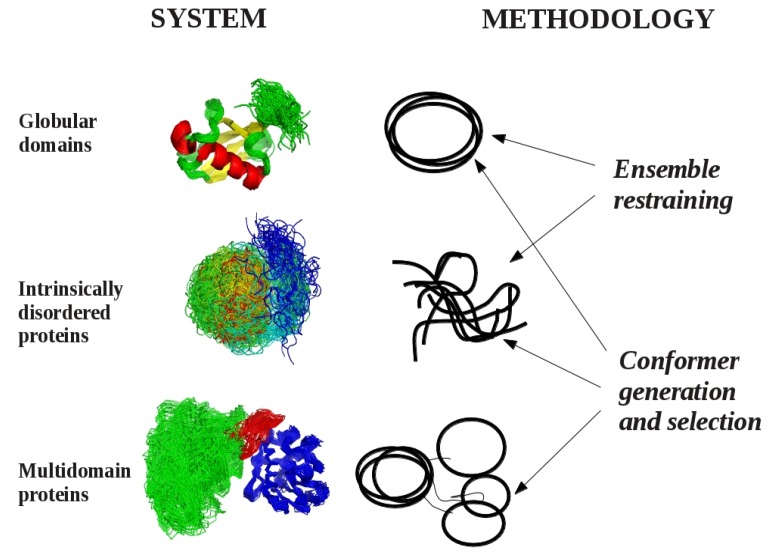
Systems and methodologies suitable for the generation of dynamic structural ensembles.

Currently we can define three different cases where dynamic structural ensembles can be relevant (Figure 1). The first corresponds to globular protein domains, proteins conventionally viewed as “well-defined” structures, where the internal motions can be reasonably approximated with fluctuations around an average structure. The most famous of such proteins is ubiquitin, a relatively rigid molecule for which a number of ensembles have been generated to study different aspects of its internal motions on time scales ranging from picoseconds to microseconds. The second category can be represented by multidomain proteins, where dynamics is realized by the multitude of possible relative orientations of rigidly treated globular domains connected with more flexible linker regions. The third class consists of intrinsically disordered proteins, for which descriptions based on average or “representative” structures are unsuitable.

## 3. Methodological Aspects of Ensemble Generation

One of the key aspects of the generation of dynamic structural ensembles is that, as described above, all NMR-based parameters are interpreted as ensemble properties. This has several consequences, namely, that no single conformer will fulfill all the parameters, making the choice of “representative conformers” harder, subjective or even unfeasible. However, the inherent dynamical aspect of NMR-derived parameters is explicitly taken into account. Last but not least, potential conflicts between NMR-parameters and the force field used are relaxed, potentially leading to better overall geometries of the members of the ensemble. Similar improvements can be expected when the experimental restraints are not entirely compatible with a single structure but reflect structural heterogeneity. In addition, parameters directly reflecting internal dynamics at a given time scale can be used as restraints, resulting in an ensemble corresponding to mobility at that time scale. It should be noted that most NMR parameters report both on structure and dynamics simultaneously.

From the methodological point of view, there are currently two major approaches that can be used to generate dynamic structural ensembles. The first one is using experimental parameters as restraints to ensure correspondence to measurements during the generation of the conformations. Restraints can be applied in an ensemble- or time-averaged manner, the former of which will be discussed in detail below. The second main approach is to generate a large pool of possible conformations and to use a selection algorithm to arrive at an ensemble that fulfills all desired parameters. The selection procedure can be deterministic or stochastic and the resulting ensembles can vary greatly in their size.

The size of the ensemble and its interpretation gives rise to several questions not without connection to science philosophy. First, it is evident that by increasing the number of conformers the degrees of freedom of the system get larger; thus, correspondence to experimental parameters is more easily achieved (overfitting). Second, we still cannot be sure that if we have an ensemble conforming to all of the desired parameters in an acceptable way, then we have a description of the actual internal motions/structural heterogeneity of the system. Thus, we should be aware that these representations, although usually yield a better description of proteins than single conformers, are still models that are not necessarily error-free.

### 3.1. Ensemble Restraining Approaches

**Figure 2:**
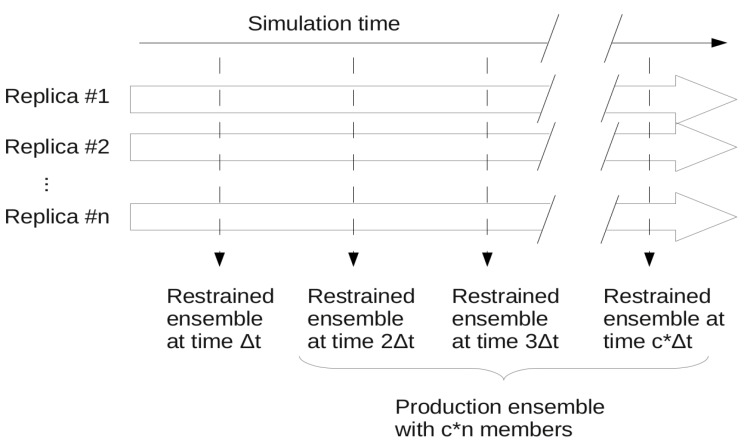
Scheme of ensemble restraining with the ensembles generated. A total of c snapshots are taken from n parallel simulations at Δt times yielding a production ensemble of c*n conformers.

In ensemble restraining, a number of parallel replicas are simulated and the applied restraints are treated as an ensemble property. The method ensures that at each simulation step where restraining is applied, the actual ensemble composed of *n* replicas corresponds to the restraints. Usually, a super-ensemble is used as final or “production” ensemble that consists of a number of synchronous ensembles taken from a given portion of the full simulation (Figure 2). There can be cases, especially when only S^2^ restraining is applied and/or the ensemble is not required to stay close to the starting structure, that a structural drift occurs during the simulation,. In this case the “production ensemble” will not necessarily reflect the restrained experimental parameters and thus should be carefully evaluated as detailed below.

As molecular dynamics simulations are deterministic, if the initial states for all of the replicas are identical, they will not be able to deviate from each other during the simulation and effectively no ensemble restraining will be present. Thus, either different starting structures should be used or the set of initial atomic velocities should be unique for each replica.

A frequent common aspect of ensemble restraining is to turn on the restraint-associated forces relatively gently, which is especially important when the initial state does not correspond well to the restraints to be introduced. One of the solutions to this is to use “half-harmonic” restraining where the restraint potential is adjusted according to how close the system already got the desired values. A progress variable ρ can be introduced, typically separately for each parameter type (Equation (1), [13]):

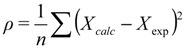
(1)
where *X_calc_* and *X_exp_* correspond to the back-calculated and measured parameters, respectively, and *n* is the number of available parameters of the given type. The minimal value of this variable reached during the simulation is then used to calculate the restraint energy *E*_*restr*_, which is nonzero only when *ρ >ρ_min_* [Equation (2)]:

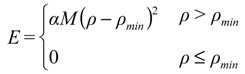
(2)
This method ensures that the systems fluctuations are directed towards states that conform to the restrained parameters better while keeping the restraining forces relatively low. In the following we overview the parameters that can be restrained in ensemble calculations and the protocols used to generate restrained ensembles.

#### 3.1.1. S^2^ General Order Parameters

General order parameters, denoted S^2^, are commonly used to describe the extent of motions on the ps-ns time scale and are usually derived from heteronuclear relaxation data [14]. They are somewhat unique among the parameters that can be restrained in the sense that as they reflect the heterogeneity of bond orientations directly, they can only be meaningfully defined on an ensemble (S^2^ parameters for a single structure are always unity). A practical aspect of S^2^ restraining is that order parameters are characteristic of the motions within the molecular reference frame, meaning that bond orientations should be compared only for superimposed conformers. The exact set of atoms for which this superposition should be done could be a matter of consideration, although most commonly all backbone atoms are used. S^2^ restraining is available in XPLOR-NIH [15] and has also been implemented as extensions to CHARMM [16] and GROMACS [17].

#### 3.1.2. NOE Distances

NOE distances are commonly used for conventional structure calculations in protein NMR spectroscopy and are prevalent examples of parameters that are usually not totally fulfilled by a set of highly similar conformers [18]. However, they can also be interpreted in an ensemble-averaged way allowing single structures to deviate remarkably from the restrained value while the distance averaged over all conformers remains consistent with the experiments [19,20]. NOE distances are averaged in a non-linear way and there is a consensus that ambiguous distance within each conformer should be averaged using the reverse sixth power of the individual distances. However, implementations differ on the treatment of inter-replica averaging, either using reverse sixth or third power. The latter is claimed to be a better approximation if the internal motions are assumed to be faster than the overall rotational reorientation of the protein [21]. NOE restraints can be particularly prone to under-restraining or overfitting, as ensembles containing a single highly deviating structure can still fulfill the restraints when distances are averaged. The ideal ensemble size for NOE restraining is considered to be as few as two replicas [18,21], and a practical implementation of this is included in the originally described version of the MUMO (minimal under-restraining minimal over-restraining) approach where NOE averaging is performed over the replicas in an overlapping pairwise manner [21]. Ensemble NOE restraining is available in XPLOR-NIH [15] and GROMACS, pairwise averaging has been implemented as in-house extension to CHARMM [21] and also in a publicly available modification for GROMACS [17].

#### 3.1.3. Residual Dipolar Couplings

Residual dipolar couplings report on the structure and dynamics of proteins up to microseconds [22]. Characterization of internal dynamics by RDCs requires at least five independent data sets obtaining which might not be feasible for all proteins and cases of interest [23]. RDC restraining requires the knowledge of the alignment of the protein in the oriented media. For this, several approaches exist depending on whether a common alignment is assumed for all replicas and how the alignment tensor itself is estimated. For example, the GROMACS implementation uses the same alignment tensor for each replica calculated so as to optimize the correspondence between back-calculated and experimental RDCs [24]. Other approaches take into account the fact that fluctuations in the molecular shape affect the alignment [25] and estimate the alignment tensor separately for each replica based on molecular shape and electrostatics [26]. Ensemble-based RDC restraining is available in XPLOR-NIH and GROMACS; the approach to consider individual alignments of the replicas has been implemented in GROMACS [27].

#### 3.1.4. Chemical Shifts

Chemical shifts (CS) have been shown to bear sufficient information to obtain correct protein folds [28]. However, using CSs in MD simulations has not been straightforward due to the fact that although methods to estimate chemical shifts from structure are available for a while [29], the expressions used in early methods were not differentiable, hindering the calculation of forces arising from this term during MD. Thus, a set of differentiable expressions had to be developed to implement this type of restraint [30], and this paved the way for the ensemble-based simulations restrained with CSs [31]. Ensemble CS restraining has been implemented in GROMACS [32].

#### 3.1.5. Other Types of Parameters

Other, less frequently used types of parameters used for ensemble calculations include distances obtained from paramagnetic relaxation enhancement (PRE) [33], scalar couplings [34,35], H-D exchange protection factors [12], and radius of gyration [36]. The importance of PRE and radius of gyration parameters is that they report on long-range interactions and the global structure of the proteins. Besides applications in solution NMR, ensemble simulation techniques for the incorporation of solid-state NMR observables have been developed and successfully applied in the study of membrane proteins [37].

#### 3.1.6. Protocols for Restraining

For most applications, multiple types of ensemble-averaged restraints are used to obtain a more complete and realistic picture of the internal motions of the protein. There are several protocols, usually identified with an acronym, that have been described in the literature in the past years. Table 1 lists some of these along with the restraint types and restraining mode they represent.

**Table 1:** Several named protocols for generating dynamic structural ensembles.

Protocol (ensemble restraining)	Parameters used and number of replicas (under parentheses)				Reference
	NOEs	S^2^ order parameters	J-couplings	RDCs	
DER (Dynamic Ensemble Refinement)	√ (8)	√ (8)			[34]
DER modified	√ (8)		√ (16)		[35]
MUMO (minimal under-restraining minimal over-restraining)	√ (2)	√ (8)			[21]
EROS				√ (8)	[38]
ERNST	√ (2)			√ (64)	[39]

It should be noted that different NMR-derived parameters average effectively on different time scales and ensemble sizes, the latter of which can also reflected in the name of the protocol. For example, the MUMO protocol, as originally described, uses eight replicas for S^2^ restraining and NOE restraining in a pairwise linked manner over the replicas. However, the term “MUMO” in general refers to considering different ensemble sizes for different restraint types rather than being the name of a strictly defined protocol.

Another issue to be considered is how the MD run itself is set up. To achieve sufficient sampling of available conformations, a standard MD run at a constant temperature might not be the best choice. For example, simulated annealing (SA) cycles can be used and the synchronous ensembles then correspond to the final step of each SA cycle. Additionally, the force constants of the restraints can be adjusted for different phases of the SA cycles [21]. Such a scheme has a high methodological resemblance to conventional NMR structure calculations.

### 3.2. Methods for Conformer Generation and Selection

For systems with considerable conformational diversity, such as multidomain or intrinsically disordered proteins, restraining protocols are not always feasible. The other possibility to obtain NMR structural ensembles is the generation of a very large pool of different conformers during the molecular simulation and then applying a conformer selection algorithm to choose the most relevant subset of conformers in accordance with available experiment-derived data.

#### 3.2.1. Conformer Generation

Possible methods for conformer generation include MD simulations and *de novo* conformer building. For both approaches, reaching maximal diversity while ensuring the geometric feasibility of the structures is a key aspect. As classical MD methods do not provide sufficient sampling, a reasonable choice for globular proteins is accelerated molecular dynamics (AMD) [40]. However, for proteins with a higher degree of conformational diversity, which is typical for intrinsically disordered proteins, sampling approaches able to cover a larger portion of the theoretically available conformational space might be required. Such methods generally rely on the use of precomputed conformer distributions of amino acid building blocks [41]. Naturally, the sampled conformational distribution should be realistic both locally, ensured by the sampling of low-energy amino acid conformations, and globally, by introducing terms to avoid spatial overlaps between residues. The flexible-meccano approach was designed specifically to generate conformational ensembles of IDPs using amino acid conformers occurring in loop regions of high-resolution crystal structures as default, although the secondary structure propensities of selected segments can be modified if needed [42]. This algorithm uses a residue-specific hard sphere model to avoid atom-atom collisions but allows the introduction of constraints describing observed long-range interactions. Another approach, called TraDES, is able to generate a wide range of polypeptide conformations ranging from native-like folded to largely unfolded ones. TraDES also allows for adjusting the sampling of residue conformations according to their characteristic occurrence in secondary structure types [43]. TraDES uses an efficient backtracking-rebuilding algorithm to eliminate overlapping conformations during model building.

A special approach of conformer generation is used for multidomain proteins where the domains can be treated as rigid bodies while probing realistic conformations of the flexible linker whilst avoiding steric clashes. Such calculations can be performed with the Pre_bunch algorithm, originally developed for the BUNCH program used to interpret small-angle X-ray scattering (SAXS) data [44] and used in ensemble selection approaches like EOM [45] and MaxOcc [46].

#### 3.2.2. Conformer Selection

Some of the early applications that can be mentioned here evaluated the whole ensemble generated against experimental data and no selection step was performed [47,48,49,50,51]. These studies proved that the ensemble approach to describe peptide and IDP structures is highly fruitful but also pointed out that such ensembles could be improved to match experimental data better by readjusting the conformational preferences of the amino acid building blocks of the protein in question [52]. In line with such considerations, the need for a selection step where only a subset of conformations is retained or structures are weighted [53] has become widely accepted. Current approaches designed for this task use ensemble Monte Carlo calculations or genetic algorithms and use a predefined maximal final ensemble size. An approach of different philosophy, MaxOcc, is also discussed briefly below.

The program ENSEMBLE uses Monte Carlo optimization to select a sub-ensemble from the “initial soup” corresponding to a set of user-generated conformations [54]. The ideal size of the initial soup is around 100,000 structures for an IDP and conformations are allowed to be selected multiple times to contribute to an initial ensemble of not more than 5,000 structures. This pool is subsequently optimized by swapping one of its constituent structures with one from the initial soup and applying a Metropolis Monte Carlo criterion to accept or reject the new ensemble according to its correspondence to experimental data. ENSEMBLE is able to handle CS, RDC, scalar coupling, R_2_ relaxation rate, paramagnetic relaxation enhancement (PRE), PRE ratio, NOE, solvent accessibility and hydrodynamic radius (R_h_) as well as SAXS data [54].

The program ASTEROIDS uses a genetic algorithm to produce and ensemble of IDPs consistent with experimental data, measured with a simple quadratic fitness function measuring the deviation of the back-calculated parameters from the measured ones. ASTEROIDS can handle diverse NMR-derived parameters like RDCs [55], PREs [56] and chemical shifts [57]. The algorithm maintains 100 different ensembles of size N. The next generation is selected from 4 × 100 novel ensembles, generated by exchange of structures either from a previously selected generation (called internal mutation) or the entire pool of conformations (external mutation), as well as crossing over (random pairing of ensembles from the previous generation) and novel sets of randomly selected structures. From these ensembles, 100 are retained using tournament selection between randomly created groups of ensembles. Details of the selection process are optimized for robustness and to avoid being trapped in local minima [55]. The approach SUPERNOVA is conceptually similar to ASTEROIDS but developed for globular proteins [58]. Genetic algorithms can also be used for assessing the distribution of relative domain orientations in multidomain proteins. In a recently developed approach, selection is based on consistency with steric RDCs (X. Salvatella, personal communication).

The MaxOcc algorithm was developed to select a set of domain orientations for multidomain proteins to fit experimental RDCs, pseudocontact shifts and SAXS data. The algorithm requires a pregenerated set of structures sampling possible domain orientations. From this set, a number of conformations will be tested with maximum occurrence (MO) analysis. The selected conformation is put into an ensemble along with others from the pool and the correspondence of the ensemble to the experimental values is checked for different weights of the selected structure. The maximal weight, corresponding to the maximal occurrence is defined as the largest value with which the experimental data are reproducible. The MO value does not mean that the conformation in question is populated to that extent, only that its contribution to the observables can not exceed it. Thus, although MaxOcc analysis reports a number of structures with MO values above a selected threshold, they can not be regarded as equivalents to the ensembles generated by the other method above.

## 4. Evaluation and Properties of Dynamic Structural Ensembles

### 4.1. Validation of Dynamic Ensembles

The ensembles obtained either by restraining or ensemble selection should be evaluated for correspondence to experimental data. The robustness of the ensemble description is commonly assessed by cross-validation, correspondence to data not used in the generation process. If a large number of data sets are available, e.g., for RDCs, leave-one-out procedures can be systematically performed [38]. In other cases, simply the parameters that are not suitable for inclusion in the given calculations can be used. It should be noted that requiring the compliance of an ensemble to all available measured parameters is probably not reasonable. Most importantly, different parameters report on motions on different time scales, thus, those not included in the calculation and averaging on time scales that are faster or slower than those used directly will not be expected to be reproduced. Moreover, the large number of parameters itself and possible limitations in our knowledge about their physical interpretation and averaging might also render such expectations impractical. The Correspondence of NMR-derived structural Ensembles to eXperimental data (CoNSEnsX) provides a simple interface to investigate the relation of structural ensembles to available experimental data [59]. By taking a PDB file with the ensemble, an X-PLOR format NOE distance list and a BMRB file it performs a simple ensemble-based evaluation of the ensemble as a whole. To back-calculate RDCs and chemical shifts, CoNSEnsX relies on PALES [28] and SHIFTX [29], respectively, and reports the correlation, RMSD and Q-factor for each parameter it can interpret and is present in the BMRB file. The server output also contains graphs that allow comparing the correspondence of each individual structure to the given parameter and that of the ensemble-averaged value. Typically, the dynamic ensemble shows better correspondence to the measured parameters than the majority of the individual structures. It is important to note that CoNSEnsX does not yield a single numerical measure of the “goodness” of the ensemble because of the consideration that an ensemble model that is only moderately more realistic than single-conformer or other ensemble representations can still be used to explain some biochemical features [59].

### 4.2. Analysis of Ensemble-Based Representations

Dynamic structural ensembles comprise a novel type of models of protein structures and their value is the addition of a fourth, dynamic dimension to our understanding of the relationships between protein structure and function. Such models exhibit properties not readily available from conventional NMR ensembles or X-ray structures. For example, burial of residues can vary greatly from conformer to conformer in such ensembles, with the most deeply buried residues showing the largest variability [60], adding a novel aspect to the role of structure-stabilizing interactions in the protein core. Analysis of such ensembles is most similar to the evaluation of molecular dynamics runs with the important difference that no trajectory is available, *i.e.*, pathways of interconversion between the obtained conformers cannot be evaluated. However, such ensembles are suitable to analyze the extent of correlated motions [39] and changes of the overall shape of the molecule and more subtle alterations in biochemically important regions such as ligand binding sites [38]. It should be noted that although parameters for side-chains can also be applied in such calculations [61], most of the NMR-derived data used in protocols restrain the structure and dynamics of the protein backbone, leaving the determination of side-chain orientations and contacts to the force field used.

Detailed analysis often requires comparison of different ensembles, e.g., those generated with different protocols, as well as dynamic ensembles versus a set of conventionally determined NMR models or different X-ray structures like complexes of the same protein with different partners. Such comparisons can be done using reduction of dimensionality like principal component analysis (PCA) [62]. More sophisticated methods treat the ensembles as samples from a distribution and can assess whether particular conformations occur at similar frequencies [63].

### 4.3. Do Dynamic Ensembles Reflect “Reality”?

Dynamic ensembles break with the common representation of protein structures with single conformers. Although the dynamic nature of proteins is widely acknowledged, the exact inclusion of dynamics in structural representations is not without discrepancies. First and foremost, reproduction of all experimental data becomes easier using ensembles of multiple conformations just because of increasing the degrees of freedom, opening up the path to possible overfitting. Overfitting or under-restraining occurs when the number of replicas is unnecessarily high and the heterogeneity of the resulting ensemble reflects uncertainty rather than the information content of the restraints. On the other hand, in the case of underfitting or over-restraining the number of replicas is not sufficient to consistently account for the dynamics reflected by the experimental information [21]. This problem is specifically addressed in the MUMO approach that allows the use of an optimal ensemble size of each parameter used in a single simulation. In each case, careful cross-validation is necessary to ensure that the resulting ensemble is neither over- nor under-restrained. It should also be considered that the number of conformations sampled by the protein during its dynamics is orders of magnitudes larger than the typical tens or hundreds of structures obtained from an ensemble-restrained run. Still, it might well be that some of the simulated conformations are not adopted by a significant fraction of the molecules in reality. What can be reliably assessed is the probability of each conformer to correspond to a given set of experimental data and our physical knowledge, as implemented in the inferential structure determination (ISD) method [64].

In ensemble generation-selection approaches the minimal number of structures suitable to reproduce all desired data is also investigated but might vary from case to case. Another related problem stems from the degeneracy of the ensembles as solutions to reproduce experimental data. The fact that we can found a set of conformations that describes our experimental data does not necessarily mean that these structures reflect the actual sampling of the conformational space of the protein [41]. To explicitly address this degeneracy, a Bayesian weighting approach was developed which can explicitly account for the uncertainty of the individual weights assigned to conformations in IDP ensembles [65]. A different approach, applicable to globular proteins, is to ensure that the ensemble does not deviate much from the single-conformer representation (e.g., an X-ray structure) while accounting for the dynamics at the same time [3].

The question whether ensemble-restrained conformer sets reflect Boltzmann distributions has been recently addressed in detail [66,67,68]. The general conclusion is that ensemble restraining is in principle capable of producing ensembles that represent a reasonable approximation of the Boltzmann distribution of the system. However, this requires that the description of the underlying physical interactions (*i.e.*, the force field) is reasonably accurate, and does not mean that routinely calculated ensemble-averaged ensembles have this property.

## 5. Alternative Approaches

To put the described methods into wider context, we briefly discuss some other approaches aimed at generating conformer ensembles that correspond to experimental data better than conventional SCR and MD methods.

### 5.1. Use of Time-Averaged Restraints

An alternative to ensemble restraining is to use time-averaged restraints. Similarly to ensemble restraining, instantaneous correspondence of a single conformer to all of the restraints is not required, rather, average values calculated from snapshots taken from a single run are expected to match the experimental observations closely. In the course of the simulation, the time-averaged parameter is calculated from the structures at previous steps with the application of an exponential “memory decay” factor. This is necessary to avoid cases where the restraint force would be zero despite an instantaneous violation but when the time-averaged value is still within the restrained boundaries [69]. Due to lack of history at the beginning of the simulation, the restraint forces are turned on slowly by gradual incrementation of the associated force constant. It is necessary to note that in this restraining scheme no strict restraining potential exists because of its dependence on time [69]. Time-averaged restraining has been described for NOE [69], J-coupling [70] and RDC [24] data.

### 5.2. Methods without Restraining

One of the disadvantages of restraining methodologies is that they do not yield a trajectory that can be used to assess the processes of conformational rearrangements. In addition, reliable experimental data should be available for restraining and parameter adjustment (e.g., restraint force constants) should be done on a case-by-case basis. Moreover, restraining may introduce perturbations in the dynamics and might lead to ensembles deviating from Boltzmann statistics (but see 4.3). Thus, efforts have been made to reproduce NMR parameters without restraining.

First of all, as MD force fields are constantly improved, agreement of “standard” MD calculations with NMR parameters is better with newer force fields than with older ones [71]. This has been explicitly shown for chemical shifts [72]. In addition, it has been shown that enhanced conformational sampling alone can lead to better correspondence to NMR parameters such as RDCs, J-couplings and chemical shifts [73,74]. A recent study has shown the use of accelerated molecular dynamics in obtaining motional descriptions at multiple time scales that are in agreement with NMR data [75].

A different approach is to modify existing force fields in order to get results more concordant with NMR data. Modifications in the dihedral potentials of the amber99ffsb force field [71] were shown to reproduce chemical shifts and RDCs better than the unmodified force field [72,76].

## 6. Example Applications

### 6.1. Partner Binding and Correlated Motions in Ubiquitin

**Figure 3:**
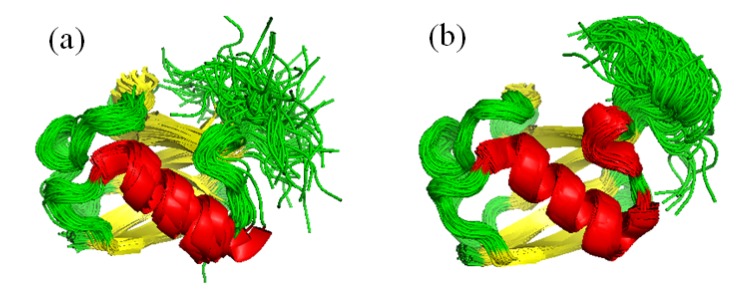
EROS (**a**) and ERNST (**b**) ubiquitin ensembles (116 and 640 conformers). The ERNST ensemble shows lower average deviation from the X-ray structure than the EROS ensemble while also reflecting internal motions up to the microsecond time scale.

Besides its wide and still increasingly recognized role in regulating biological processes, the 76-residue ubiquitin is the most widely studied globular, folded protein by NMR. Most of the dynamic structural ensemble calculation protocols were tested on this molecule, mostly because of the vast amount of experimental NMR data available for this protein. The structural ensemble generated with the EROS protocol reflects internal motions in the molecule up to the microsecond time scale and covers a larger conformational space than the MUMO ensemble corresponding to ps-ns dynamics [21,38]. Moreover, comparison of members of the EROS ensemble to available structures of ubiquitin complexes suggested a conformer selection mechanism for partner binding as the conformational space covered by the microsecond dynamics of ubiquitin in free from in solution covers that of the available complexed structures. The ERNST ensemble, described more recently, revealed collective motions in ubiquitin linking ligand binding sites more than 10 Å apart in the structure [39] (Figure 3).

### 6.2. Small, Canonical Serine Protease Inhibitors

**Figure 4:**
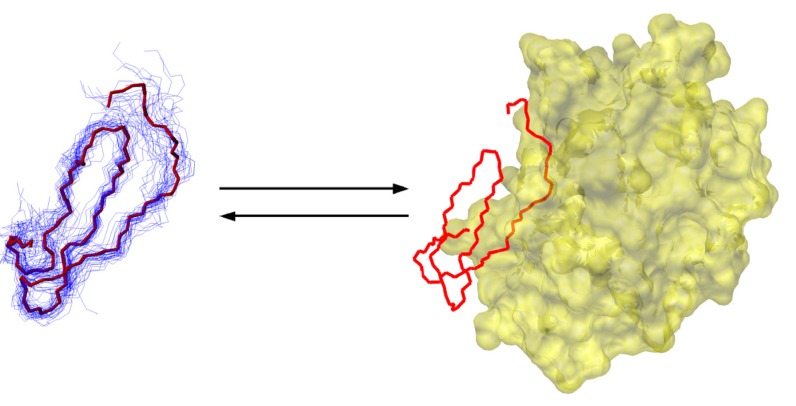
Scheme of protease binding of a small protease inhibitor highlighting its internal dynamics in the free from. The bound conformation is highlighted in red.

Canonical, standard mechanism serine protease inhibitors are classic examples of the lock-and-key molecular recognition. The textbook view of their efficiency is that the key to their efficacy is their rigidity, and no changes in the structure and dynamics of their protease binding loop occurs upon binding to the target protease. However, NMR dynamics studies from the 1990s suggested that the protease binding loop of practically all of the investigated, evolutionarily unrelated inhibitors exhibit relatively low S^2^ order parameters compared to well-folded globular parts of proteins. Dynamic ensembles of small, 35-residue inhibitors were calculated using a protocol resembling the original MUMO approach [5]. Despite exhibiting much larger conformational variability than SCR structures, a key residue-residue interaction was more constrained in the dynamic ensembles than in the conventional ones This shows that the dynamically restrained ensembles are not simply over-relaxed versions of the SCR structures and that also supports the importance of these interactions.Comparing the dynamic ensembles with complexed crystal structures suggested that the ps-ns timescale solution-state dynamics covers the enzyme-bound state of the inhibitors. Because enzyme binding is estimated to occur on a much slower timescale and thus conformational rearrangements are not expected to be limiting from the inhibitor’s side, this scenario is largely reconcilable with the rigid binding model (Figure 4).

### 6.3. The Intrinsically Disordered Protein ACTR

The intrinsically disordered protein ACTR (activator for tyroid hormone and retinoid receptors) acts as a transcriptional activator upon hormone binding by nuclear receptors. It recruits a number of partner proteins to form a complex involved in histone acetylation. ACTR and its complex with the nuclear coactivator binding domain of CREB binding protein is a frequently used model of the folding and binding mechanism characteristic of IDPs. It is likely that the binding interaction requires a preorganized conformation of ACTR in accordance with a conformer selection scenario. A recent study on ACTR combining mutagenesis studies and ensemble description based on PREs, RDCs and chemical shifts suggested the presence of long-range helix-helix interactions in the protein. Ensembles with structures containing helical segments of various length were generated with the flexible-meccano algorithm and selection was performed with the ASTEROIDS method. The significance of the obtained results lies in establishing a connection between short- and long-range interactions in a disordered protein that is not trivially detectable because of the masking effect of stronger interactions contributing dominantly to NMR observables [77].

### 6.4. The Transmembrane Helix of Vpu

An ensemble-based dynamics approach using solid-state NMR parameters (termed SSNMR-ED) was developed and applied for a transmembrane helix of the viral protein “u” (VpuTM). The main parameters investigated were the tilt and rotation angles of the helix within the membrane. The combined study of restrained and standard molecular dynamics suggested that the TM helix exhibits a considerably higher orientational variability than previously anticipated. The motion appears to be fast at the NMR time scale. The results shed light on the intramembrane mobility of TM helices which are considered important in defining distinct functional states of transmembrane proteins [78].

## 7. Conclusions

In this review we gave a brief overview of presently available approaches to generate structural ensembles of proteins that reflect their internal mobility. Such ensembles give a more realistic description of protein behavior than static structures and can be used to draw conclusions on biologically relevant processes in dynamic systems at atomic resolution using data from NMR experiments. One of the challenges of the near future is the characterization of protein complexes and their formation by detailed atomic-level description of the dynamics of the partners both in free and bound forms. Another task to solve is the generation of ensembles reflecting dynamics on multiple time scales, which can already be successfully addressed by molecular dynamics methods with enhanced sampling [75]. From the technical point of view, a number of programs and protocols have been already described, novel ones are being continuously developed and more and more become available to the structural biology community for routine use. Thus, we believe that the use of dynamic ensemble representations will get more widespread in the near future.
